# Oseltamivir Is Adequately Absorbed Following Nasogastric Administration to Adult Patients with Severe H5N1 Influenza

**DOI:** 10.1371/journal.pone.0003410

**Published:** 2008-10-15

**Authors:** Walter R. J. Taylor, Bui Nghia Thinh, Giang Thuc Anh, Peter Horby, Heiman Wertheim, Niklas Lindegardh, Menno D. de Jong, Kasia Stepniewska, Tran Thuy Hanh, Nguyen Duc Hien, Ngo Minh Bien, Ngo Quy Chau, Annette Fox, Nghiem My Ngoc, Martin Crusat, Jeremy J. Farrar, Nicholas J. White, Nguyen Hong Ha, Trinh Thi Lien, Nguyen Vu Trung, Nicholas Day, Nguyen Gia Binh

**Affiliations:** 1 Oxford University Clinical Research Unit, Hanoi, Vietnam; 2 Centre for Vaccinology and Tropical Medicine, Churchill Hospital, Oxford, United Kingdom; 3 Bach Mai Hospital, Hanoi, Vietnam; 4 Mahidol Oxford Research Unit, Bangkok, Thailand; 5 Oxford University Clinical Research Unit, Ho Chi Minh City, Vietnam; 6 National Institute for Infectious and Tropical Diseases, Hanoi, Vietnam; University of New South Wales, Australia

## Abstract

In the absence of a parenteral drug, oral oseltamivir is currently recommended by the WHO for treating H5N1 influenza. Whether oseltamivir absorption is adequate in severe influenza is unknown. We measured the steady state, plasma concentrations of nasogastrically administered oseltamivir 150 mg bid and its active metabolite, oseltamivir carboxylate (OC), in three, mechanically ventilated patients with severe H5N1 (male, 30 yrs; pregnant female, 22 yrs) and severe H3N2 (female, 76 yrs). Treatments were started 6, 7 and 8 days after illness onset, respectively. Both females were sampled while on continuous venovenous haemofiltration. Admission and follow up specimens (trachea, nose, throat, rectum, blood) were tested for RNA viral load by reverse transcriptase PCR. *In vitro* virus susceptibility to OC was measured by a neuraminidase inhibition assay. Admission creatinine clearances were 66 (male, H5N1), 82 (female, H5N1) and 6 (H3N2) ml/min. Corresponding AUC_0–12_ values (5932, 10,951 and 34,670 ng.h/ml) and trough OC concentrations (376, 575 and 2730 ng/ml) were higher than previously reported in healthy volunteers; the latter exceeded 545 to 3956 fold the H5N1 IC_50_ (0.69 ng/ml) isolated from the H5N1 infected female. Two patients with follow-up respiratory specimens cleared their viruses after 5 (H5N1 male) and 5 (H3N2 female) days of oseltamivir. Both female patients died of respiratory failure; the male survived. 150 mg bid of oseltamivir was well absorbed and converted extensively to OC. Virus was cleared in two patients but two patients died, suggesting viral efficacy but poor clinical efficacy.

## Introduction

The number of anti influenza drugs is currently limited to oral amantadine, rimantadine, oseltamivir and inhaled zanamivir and there are no registered parenteral formulations. The need for effective anti influenza drugs has been heightened by the emergence of highly pathogenic, H5N1 influenza A infection that characteristically causes a severe, rapidly progressive pneumonitis with a mortality rate of between 60 to 80%.[1], [2], [3], [4], [5] Fear of a possible H5N1 pandemic has prompted many countries to stockpile oseltamivir.

Oseltamivir phosphate (OP) is licensed for the prophylaxis and treatment of uncomplicated human influenza A and B. The active metabolite, oseltamivir carboxylate (OC), inhibits the influenza virus neuraminidase from destroying host cell sialic acid receptors, thereby preventing the release and spread of newly formed virions from epithelial cell surfaces.[6] Early use (≤48 h) oseltamivir reduces illness duration and secondary bacterial infections in influenza A and B infected adults and children.[7]

The World Health Organisation (WHO) recommends standard dose (75 mg bid in adults) oseltamivir as first line treatment against H5N1 infection with advice that clinicians consider 150 mg for severely ill patients with pneumonitis. Observational data suggest oral oseltamivir is better than no treatment and that surviving H5N1 is more likely if given early.[2], [8] However, there have been no prospective studies, no published pharmacokinetic (PK) data in H5N1 infected patients and mortality remains high in oseltamivir treated H5N1 patients.

Oseltamivir, up to 500 mg bd, is well tolerated; the main side effects have been limited to headache, nausea and mild vomiting.[9] Mean steady state (≥48 h) trough OC concentrations (Cmin) in mild influenza patients and healthy subjects are ∼200 and ∼300 ng/ml with 75 and 150 mg bid, respectively; the corresponding mean AUC_0–12_ values are 2270 and 4900 ng.h/ml.[9], [10], [11], [12] OC is filtered and secreted into the urine via the human organic anion transporter 1; doses should be halved in patients with a creatinine clearance (C_CR_Cl) of <30 ml/minute. The half life ranges from 6 to 10 hours in normal individuals and increases to approximately 36 hours in patient with end stage renal failure.[13] There are recent dose recommendations (Roche Product Information Sheet, 2007) for patients on chronic haemodialysis and chronic ambulatory peritoneal dialysis (CAPD). PK data are lacking in patients on continuous venovenous heamofiltration (CVVH) but it has been shown *in vitro* that OC crosses the haemofilter freely and has a sieving coefficient (Sieving coefficient = OC concentration in the ultrafiltrate/mean OC concentration of the haemofilter arterial and venous limbs) of 1, consistent with its polar nature and low protein binding (<3%); therefore, the CVVH ultrafiltration rate approximates to the glomerular filtration rate (GFR).[14]

The PK PD (pharmacodynamic) relationships in H5N1 are unknown. *In vitro*, mean IC_50_ OC values against H5N1 vary by viral clade and methodology, ranging from ∼0.5 (0.14 ng/ml) to 11.45 nmol/ml (3.19 ng/ml) for clades 2 and 3 and 0.09 (0.03) to 2.3 (0.8) nmol/ml (ng/ml) for clade 1.[15], [16] These values are many fold lower than OC plasma concentrations in mild influenza but the clinical efficacy of oseltamivir alone in H5N1 is limited.[1], [2], [3] Reasons for this may include irreversible lung damage at presentation, immune mediated pathology, development of H5N1 resistance to OC, reduced OC tissue distribution/penetration and low oseltamivir absorption in severely ill patients secondary to possible gastric paresis, small bowel ileus or diarrhoea.[5], [17] Better insight into the pharmacokinetics and pharmacodynamics of OC in patients with H5N1 and other forms of severe influenza is urgently needed.

Herein, we report the PK of nasogastrically administered oseltamivir 150 mg bid in three severely ill patients with H5N1 (n = 2) and H3N2 influenza who were also treated with CVVH.

## Methods

The patients, a 30 years old male (A), and a 22 years old, 7 months pregnant female (B) with H5N1 influenza and one 76 years old female (C) with H3N2 influenza, were admitted to the intensive care unit (ICU) of the Bach Mai hospital, Hanoi, Vietnam. Written informed consent was obtained from patients or their relatives. The study was approved retrospectively by the Bach Mai hospital Ethics Committee because of the importance of studying rapidly such patients in the absence of a prospectively approved protocol.

Virological diagnosis and viral load measurements were done by reverse transcriptase (RT) PCR on nasal, throat and rectal swabs, endotracheal aspirates, pleural fluid and plasma.[18] The lower limit of viral load detection is 1000 cDNA/ml. All respiratory specimens were inoculated onto Madin Darby Canine Kidney cells for influenza virus isolation. *In vitro* susceptibility of virus isolates to OC were tested by a chemiluminiscent neuraminidase inhibition assay (NA-Star, Applied Biosystems).

All patients were prescribed oseltamivir (Tamiflu®, Roche, Basel, Switzerland) 150 mg bid (double dose)×10 days for presumptive H5N1; they were dosed 12 hourly at 3 pm and 3 am. When the patients were ventilated, the powder in the capsule was dissolved in 20 mls of water, injected down the NG tube, followed by a 10 ml sterile water flush. Oseltamivir solution is not available in our hospital. Enteral feeding (Ensure Gold® 250 mls 3 hourly) was given daily from 6 am to 9 pm. All patients underwent CVVH (AN69 haemofilter, Rospal, France) at 45 mls/kg/hour in accordance with local practice. Supportive treatment was given as clinically indicated e.g. CVP guided intravenous fluids, cardiovascular support with adrenaline, noradrenaline, dobuatamine, dopamine, chest drain for pneumomediastnum and pneumothorax.

For PK measurements (predose, 05 h, 1 h, 1.5 h, 2 h, 5 h, 7 h, 9 h, 11 h, 12 h post dose), 2 ml of whole blood were collected into fluoride-oxalate tubes [19], [20] via a venous catheter (Patient A) or the arterial and venous limbs of the haemofilter (only Patients B & C were sampled on haemfiltration) ≥48 hours after starting oseltamivir when all patients were receiving NG oseltamivir. The ultrafiltrate (haemofilter exit port) was collected from Patient B. Plasma was separated by centrifuging at 2000 g for 4 minutes and stored at −80°C until analysed by using: (i) mixed mode (MPC-SD, 3 M Empore Bracknell, UK) solid phase extraction and hydrophilic interaction liquid chromatography mass spectrometry (HILIC-LC-MS/MS), (ii) an API 5000 triple quadrupole mass spectrometer (Applied Biosystems/MDS SCIEX, Foster City, USA) equipped with a TurboV™ ionisation source interface in positive ion mode, (iii) an Agilent 1200 liquid chromatographicsystem (Agilent technologies, Santa Clara, USA) and (iv) Analyst 1.4 (Applied Biosystems/MDS SCIEX, Foster City, USA).[21] The coefficients of variation (CV%) were 5.1%, 2.6% and 4.6% at 3 ng/mL, 20 ng/mL and 150 ng/mL, respectively, for oseltamivir and 3.7%, 3.4% and 4.0% at 30 ng/mL, 400 ng/mL and 4000 ng/mL, respectively, for OC.

The OP and OC steady state area under the curve values over 12 hours (AUC_0–12_) were calculated using the trapezoidal rule (Stata version 9) and clearances calculated as dose/AUC_0-τ_, (τ = dosing interval). Estimated clearance is the apparent clearance CL/f where f is the fraction of osetlamivir absorbed and converted to OC. The haemofilter clearance (ml/min) of OC over the 12 hours dosing period was calculated using the formula UV/P (U = ultrafiltrate concentration, ultrafiltrate volume, P = plasma concentration).

## Results

All patients were severely ill requiring mechanical ventilation and treatment with inotropes/vasopressors within 24 hours of ICU admission (Table 1). Patient C was in renal failure. The CVVH ultrafiltration rates (L/h) and their approximate, equivalent GFRs (ml/min) were 2.75 L/h [45.8 ml/min (patient A)], 2.2 L/h [36.7 ml/min (patient B)] and 2 L/h [33.3 ml/min (patient C)]. All patients received intravenous antibiotics for possible bacterial pneumonia and intravenous hydrocortisone (300 mg daily). Patients A and C developed pneumomediastina and bilateral and unilateral pneumothoraces, respectively, while ventilated.

**Table 1:** Patients' clinical, pharmacokinetic and virological features.

Patients	A: Male, age 30 H5N1	B: Pregnant female, age 22 H5N1	C: Female, age 76 H3N2
Duration of illness in days pre ICU	8	6	7
Poultry exposure	Y	N	N
Fever	Y	Y	Y
Cough & shortness of breath	Y	Y	Y
Vomiting	Y	N	N
Diarrhoea	N	Y	N
Weight kg	61	47.5	46
Temperature °C	40.1	38.2	39
Hb g/dL	15.5	10.3	8.3
Total white cell (lymphocyte) count /µL	5,600	5,400	16,800
Absolute lymphocyte count /µL	400	300	1680
Platelet count /µL	87,000	119,000	125,000
Serum Creatinine µmol/L	128	73	499
Creatinine clearance mls/min*	66	82	6
FI0_2_, pH, PO_2_, PCO_2_, HCO_3_^−^ ¶	40%, 7.5, 34.9, 40.4, 30.9	32% 7.45, 42.7, 28.6, 19.8	50%, 7.43, 33.3, 28, 19
Day of ICU admission	D0	D0	D0
CVVH started (start-stop Day)	D4 to 7	D1 to 4	D1 to 8
Oseltamivir started (ICU Day)	D0	D1	D2
Oseltamivir started from day of illness onset	8	7	9
Viral load cDNA copies/ml (Day measured)	3.12×10^5^ (D3)	1.14×10^5^ (D2)	2.79×10^4^ (D3)
PK sampling	D8	D3	D5
Cmin OC ng/ml#	376	575	2730
Cmin OP ng/ml#	5.49	37.4	24.4
Cmax OC ng/ml	591	1210	1270
Cmax OP ng/ml	122	156	28.7
OC AUC_0–12_ ng.h/ml	5,932	10,951	34,670
OP AUC_0–12_ ng.h/ml	395	1059	628
OC oral clearance L/h	27.8	15.1	4.7
OP oral clearance L/h	380	141.5	238.7
Outcome (Day of death)	Survived	Died (D4)	Died (D8)

* Cockroft Gault formula.

¶ Arterial blood gas prior to ventilation, mmHg: PO_2_, PCO_2_, mmol/L:HCO_3_^−^.

# OP = oseltamivir phosphate, OC = oseltamivir carboxylate.

Patients B and C died of progressive respiratory failure 10 and 14 days after illness onset, respectively. Their antemortem chest X rays had worsened since admission and showed progression from unilateral to bilateral consolidation (A) and progressive bilateral consolidation (C). Patient A was mechanically ventilated for 6 days and survived. He complained of severe headache and had persistent vomiting; consequently, his oseltamivir was stopped after 8 days of treatment.

**Figure 1:**
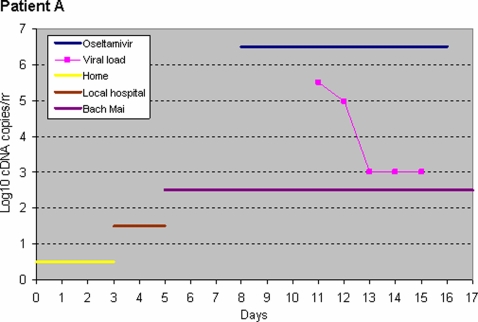
Viral clearance in patients A. Day 0 is the day of illness onset.

**Figure 2:**
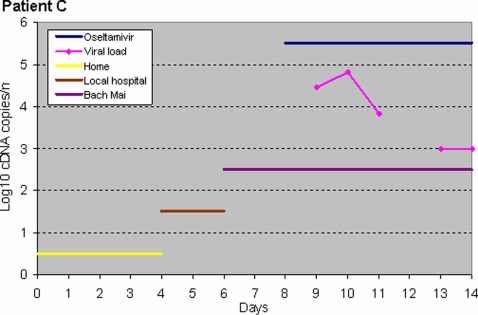
Viral clearance in patient C. Day 0 is the day of illness onset.

For viral specimen collection, Patient A was first sampled three days after starting oseltamivir; only his tracheal aspirate was positive and became negative after 5 days of oseltamivir treatment (Figure 1). Patient B was sampled once, one day after starting oseltamivir; her tracheal aspirate (1.14×10^6^ copy DNA copies/ml), pleural fluid (2.66×10^4^), stool (3.2×10^4^) and plasma (7.29×10^3^) were H5N1 positive. Patient C, sampled one day after starting oseltamivir, cleared H3N2 virus from her nose swabs after five days of treatment (Figure 2). Virus could not be isolated from specimens of Patients A and C, possibly due to the effects of oseltamivir. The *in vitro* IC_50_ of the isolate from Patient B (tracheal aspirate) was 2.5 nM (0.69 ng) per mL.

**Figure 3:**
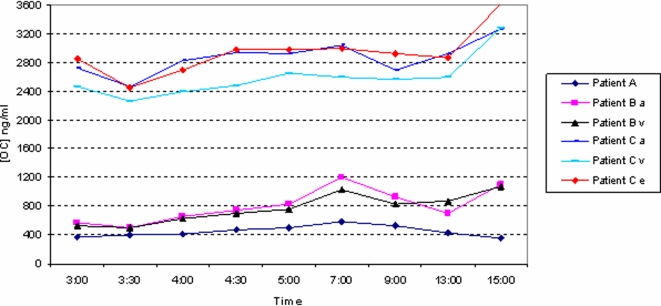
Oseltamivir carboxylate (OC) concentrations taken from the arterial (a) limb of the haemofilter (from the patient) and the venous (v) haemofilter (to the patient). For clarity, the OC concentrations from the exit limb (e) to the effluent bags are shown for Patient C only. Sampling for Patient A was off the haemofilter.

Trough OC concentrations varied markedly: 376 (A), 575 (B) and 2730 (C) ng/ml and were consistent with the steady state AUC_0–12_ and apparent oral clearance values (Figure 3 & Table 1). These OC concentrations were 545 and 833 fold higher than the IC_50_ value from the H5N1 virus isolated from Patient B and 3042 fold higher than the reported mean IC_50_s for H3N2 viruses (up to 0.9 ng/ml). The mean OC sieving coefficients were 1.055 (Patient B) and 1.1 (Patient C). Oseltamivir and OC haemofilter ‘excretions’ were 1.4 and 25 mg over 12 hours for haemofilter clearances of 25.19 and 41.46 ml/min, respectively.

## Discussion

This study has shown that in ventilated patients with severe influenza, NG administered, double dose oseltamivir was absorbed and converted extensively to oseltamivir carboxylate. The OC trough and peak concentrations were higher those reported in healthy volunteers and were many fold higher than the reported H5N1 and H3N2 IC_50_s. However, two patients died despite these good PK parameters.

The current WHO guidelines suggest clinicians should consider a double dose oseltamivir in severely ill patients because of the uncertainty of oseltamivir absorption and the high disease mortality. At the time of the study, there were no recommendations for patients on any form of renal replacement therapy from the WHO or the manufacturer but there were PK data for patients on chronic haemfiltration and CAPD[13] that have now been incorporated in the manufacturer's product information sheet. We treated our patients presumptively with double dose oseltamivir, including Patient C with the low creatinine clearance, because we suspected they had H5N1, standard dose oseltamivir for H5N1 is associated with a poor outcome in Viet Nam, and elsewhere, and to guard against the unknown loss of OC through the haemofilter.

In the event, all patients had OC concentrations and AUC_0–12_ values that were 1.3 to 9 and 1.2 to 7 times higher than reported in mild influenza/healthy volunteers, respectively, and are probably related to the impaired renal function in our patients. At the ultrafiltration rate used, the OC haemofilter loss was small, 25 mg (∼20–23% of the estimated bioavailable oseltamivir dose [9]) and does not warrant a compensatory dose increase. The H5N1 viral loads in our patients were consistent with those (log_10_ cDNA 4.3–8.2) from another Vietnamese series.[5]

The surviving patient started oseltamivir eight days into his illness. His viral clearance time of 5 days post treatment is similar to earlier observations.[5] When off the ventilator, he complained of severe headache and vomiting so oseltamivir was stopped, with good effect, after 8 days, two days short of the recommended extended dose of 10 days. We believe these symptoms were oseltamivir related which suggests his OC concentrations were high and not well tolerated.

The pregnant patient had plasma OC concentrations over the 12 hours, exceeding ∼833 fold the IC_50_ of her infecting H5N1 virus. Her calculated C_CR_Cl (82 mls/min) is probably overestimated given the dilutional effect of pregnancy. She had two poor prognostic factors: she was treated late, 7 days after illness onset, and had disseminated disease.[2], [22] Despite the susceptibility of her virus, she died of progressive respiratory failure. The lack of sequential viral load data preclude a conclusion regarding the antiviral efficacy of oseltamivir.

The elderly female had the highest AUC_0–12_ and OC concentrations. Her trough OC concentration was ∼3000 to 6000 fold higher than the reported mean IC_50_ values (0.46 and 0.9 ng) for wild type H3N2 isolates but only ∼2 to 60 fold higher than the mean IC_50_ values (1076 ng, 43.6 ng) for some mutant H1N1 and H3N2 viruses.[23], [24] Although she cleared her virus after 5 days of treatment, she died of respiratory failure. Her CVVH GFR of ∼33 ml/min was slightly above the 30 ml/min cut off for halving the oseltamivir dose, justifying our initial decision to give her double dose oseltamivir. Her OC Cmin is 2.7 fold higher than the mean Cmin (1000 ng/ml) and slightly higher than the mean Cmax (2458 ng/ml) achieved after 500 mg bd of oseltamivir.[12] Given these high OC concentrations, and our knowledge that CVVH at the rate we used results in a small loss of OC via the filter, we could have given her standard dose oseltamivir and still seen viral clearance.

There were limitations to our study. Viral sampling was incomplete and the plasma PK data were only collected once over 12 hours. We did not measure OC from the lungs, the main site of the pathology. Such data are needed to define more precisely the PK PD relationships in severe influenza and to determine a therapeutic range for patients with H5N1.

However, our data remove some of the uncertainty regarding nasogastrically administered oseltamivir. 150 mg bid of oseltamivir was tolerated, absorbed, and provided adequate plasma concentrations to arrest viral replication in ventilated, severely ill influenza patients on 45 ml/kg/h of CVVH. Nevertheless, two patients died. This may be due to late treatment and advanced disease at presentation when viral or immunologically mediated tissue pathology might be irreversible. Whether higher oseltamivir dosages, the use of antiviral combinations or adjunctive treatment might improve the outcome in patients with H5N1 influenza or other forms of severe influenza requires further research.
